# You Have My Word: Reciprocity Expectation Modulates Feedback-Related Negativity in the Trust Game

**DOI:** 10.1371/journal.pone.0119129

**Published:** 2015-02-26

**Authors:** Qingguo Ma, Liang Meng, Qiang Shen

**Affiliations:** 1 School of Management, Zhejiang University, Hangzhou, China; 2 Neuromanagement Lab, Zhejiang University, Hangzhou, China; 3 National Key Laboratory of Cognitive Neuroscience and Learning, Beijing Normal University, Beijing, China; National University of Singapore, SINGAPORE

## Abstract

Promise is one of the most powerful tools producing trust and facilitating cooperation, and sticking to the promise is deemed as a key social norm in social interactions. The present study explored the extent to which promise would influence investors’ decision-making in the trust game where promise had no predictive value regarding trustees’ reciprocation. In addition, we examined the neural underpinnings of the investors’ outcome processing related to the trustees’ promise keeping and promise breaking. Consistent with our hypothesis, behavioral results indicated that promise could effectively increase the investment frequency of investors. Electrophysiological results showed that, promise induced larger differentiated-FRN responses to the reward and non-reward discrepancy. Taken together, these results suggested that promise would promote cooperative behavior, while breach of promise would be regarded as a violation of the social norm, corroborating the vital role of non-enforceable commitment in social decision making.

## Introduction

Even in the early human society, some basic forms of cooperative agreements already existed to maintain prosocial connections like trust and cooperation [1]. As one of these primitive agreements, promise is expressed orally and is non-binding in nature, which aims to convey the information that one is trustworthy and reliable to other partners in social interactions [2]. Despite its non-enforceable nature, in the contemporary society, a large number of social exchanges still rely on such oral commitments, mainly due to its simple, valid and efficient features.

In the field of behavioral and experimental economics, trust game (TG) was designed to investigate people’s trust and cooperative behaviors as well as various factors contributing to the trust of investors. Therefore, trust game can well be adopted to examine the influence of promise on cooperative behaviors as well as its effect on outcome evaluation subsequent to the action of keeping or breaking the promise. Trust game, initially proposed by Berg et al. [3], is a one-shot game between two anonymous players, an investor and a trustee. The investor is firstly assigned with some tokens, and can choose to keep all of them or invest some of the endowment to the trustee. The tokens invested to trustee are multiplied. Then the privilege goes to trustee who can choose to keep all the multiplied tokens or pay certain amount back to the investor.

In the one-shot TG experimental setting, factors such as reputation, revenge and punishment do not come into play to affect any players’ monetary payoffs in a direct manner. Thus, theoretically, there is no economical reason for a rational trustee to reciprocate. Based on such a belief, the Nash equilibrium is that the investor chooses to keep all the endowed tokens and not to invest them. However, contrary to the prediction of classical game theory, previous studies showed that, most of the investors did invest considerable amounts, and many trustees did manifest certain degree of reciprocity [4]. Therefore, we can safely conclude that social preference factors like trust and trustworthiness might play vital roles in such scenarios. Until now, a large number of studies from both behavioral economics and neuroeconomics have made great efforts to investigate the behavioral and neural underpinnings of trust and trustworthiness [5,6].

As mentioned above, promise, as an important mechanism to foster trust, may bring interesting phenomena to be explored in trust game settings. On the other side, promise, non-binding by its nature, may not only be kept, but also be breached. Therefore, it’s an open and intriguing question to investigate how investors will respond to the orally non-binding commitment from trustees. Additionally, the brain mechanisms involved in such non-binding cooperative agreements also remain to be further clarified. To resolve these elusive issues, one pioneering neuroimaging study has examined the neural correlates of promise keeping and promise breaking from the perspective of trustees [1]. It is discovered that breach of promise leads to increased activation in the dorsal lateral prefrontal cortex (DLPFC), anterior cingulate cortex (ACC) and amygdala, which indicates that breaking the promise involves an emotional conflict over social norm obedience. In addition, the breach of promise can be predicted by brain activation in anterior insula, ACC and inferior frontal gyrus during promise making, suggesting that malevolence can be reflected in the brain pattern long before the action actually takes place. However, up to now, rare do we know about how sticking to the promise and violation of it would be evaluated and experienced from the perspective of investors.

In the present study, we modified the one-shot trust game and named it as promise-trust game (promise-TG). For the purpose of capturing the subjective evaluation of trustees’ commitment and how it affects investors’ subsequent investment behavior, we mainly focused on the role of investors. The major difference between the task in the current study and the classical trust game lies in the message-leaving stage, which is implemented before the investor decides whether to invest or keep the initial tokens on that round [1,7]. At the message-leaving stage, the trustee can either make a promise by leaving the message “I promise to give back half of the money.” or give up the message-leaving right. In the latter case, “The other person did not leave a message.” would be informed to the investor. In addition, to make the experimental design simple and concise, we adopted a dichotomic design for monetary parameters in the current study. To be specific, the investor has only two options to choose from. He/she may either invest or keep the entire tokens on each round. The trustee only has two alternatives as well, and can freely decide whether to keep or to break a promise. Decisions of both exchange partners will be implemented and thus cause monetary consequences. Behaviorally, we predict that the simple non-binding promise that comes from trustees would increase the expectation of the investors to receive refund and thus increase the likelihood to invest to their counterparts accordingly.

In the current study, we adopted Event-related Potentials (ERPs) to track the temporal dynamics of brain activity during outcome evaluation resulted from the fulfillment of commitment and the breach of promise. FRN is the negative deflection peaking around the 250–350 ms period upon feedback presentation, which shows maximum amplitude over medial frontal cortex. Because FRN is often found to reflect various aspects of the outcome, especially outcome valence, it is adopted to examine reward processing as the result of reciprocity or non-reciprocity.

Given the important role of FRN in the decision-making, there are two popular theories to account for its significance in reflecting the underlying mechanism of outcome evaluation, which are reinforcement learning theory and the motivational theory. According to the reinforcement account of FRN, unexpected losses would induce relatively larger FRNs than gains, which has been widely replicated in the past decade [8]. In the social domain, since equal split of assets is accepted as a long-established social norm, unfair offers are unexpected and will elicit more pronounced FRN than fair ones in the ultimatum game [9–11]. In addition, existing literatures showed that various factors might have an influence on the subjective expectancy [12,13]. For example, in one of our previous studies, we discovered that effort strengthened the expectation toward positive outcomes, and violation of this stronger expectancy led to a larger FRN deflection [14]. Given the prediction of the reinforcement theory, in the current study, when trustees made a promise at the message-leaving stage, investors’ expectancy toward trustees’ reciprocity might increase. Thus, when this expectancy was violated as reflected by the breach of promise, a stronger prediction error might occur, which might give rise to a stronger FRN deflection.

On the other hand, in term of the motivational account of the FRN, motivational significance of the outcome could explain the FRN discrepancy toward reward and non-reward in risky decision-making [12,14,15], and outcomes that are more motivationally significant would lead to enhanced FRN discrepancy, termed as differentiated FRN (d-FRN) which is the FRN elicited by losses (or negative feedback) minus that elicited by gains (or positive feedback). For instance, in one of our recent studies [16], we investigated how interpersonal relationship modulates people’s empathic responses to others’ financial gains and losses. We observed that, when subjects passively observed others executing the gambling task, d-FRN elicited toward their friends’ gain loss discrepancy was enhanced than that toward strangers, which is consistent with the increased motivational relevance exerted toward the socially closer counterparts. In a similar manner, in the current study, compared with the non-promise condition, outcomes in the promise condition bear more motivational significance to investors, and they are more concerned with whether the promise was kept or broken in the latter case. Therefore, we would predict that promise might enlarge the amplitude of the FRN discrepancy at the feedback stage.

## Methods

### Participants

Eighteen healthy, right-handed subjects aged 18–26 years (M = 22.65 years, SD = 2.29 years) participated in this study, 11 of which were male. Subjects in the present study were registered students of Zhejiang University who had normal or corrected-to-normal vision, all of whom reported no history of neurological disorders or mental diseases. This study was approved by the Institutional Review Board of Zhejiang University Neuromanagement Lab. Written informed consent forms were obtained from all subjects before the implementation of the experiment. Data from one subject was discarded because of excessive recording artifacts. Thus, data from 17 valid subjects went into the final data analysis.

### Experiment Procedure

The subjects were comfortably seated in a dimly lit, sound-attenuated and electrically shielded room. The stimuli were in text format, and were designed and presented using E-Prime software package (Psychology Software Tools, Pittsburgh, PA, USA). They were presented at the center of a computer screen at a distance of 100 cm with a visual angle of 8.69°×6.52°(15.2 cm × 11.4 cm, width × height). Subjects were instructed to use the keypad to make their choices. The experiment consisted of 4 blocks, each containing 60 trials. Participants were informed the rules of promise-TG before the experiment started. They were convinced that we had collected the promise and reciprocation decisions from 240 anonymous partners in a behavioral study and they would play non-real time with them in the ERP experiment. In order to guarantee that the act of giving a promise offers no valuable prediction for the trustees’ reciprocation, reward/non-reward outcomes were determined in a pseudorandom order, with half of the trials returning the favor while the other half in a non-reciprocity manner in both promise and non-promise conditions.

Prior to the experiment, participants were informed that they would take part in a game in which they would make a decision of whether or not to cooperate with anonymous partners. The partners were given the opportunity to make a promise to the participants before the investment decisions were made. According to our manipulation, half of the partners made promises while the others did not leave messages to the subjects. On each trial, participants were given ¥2, and they could either keep it all or invest it all. In the latter case, the partner would receive ¥10. Then the anonymous partners could either keep the entire ¥10 or give half of it (¥5) back to participants. The total amount of money could be increased by such investment behaviors. Participants were offered remuneration equal to the amount accumulated at the end of the game.

**Fig 1:**
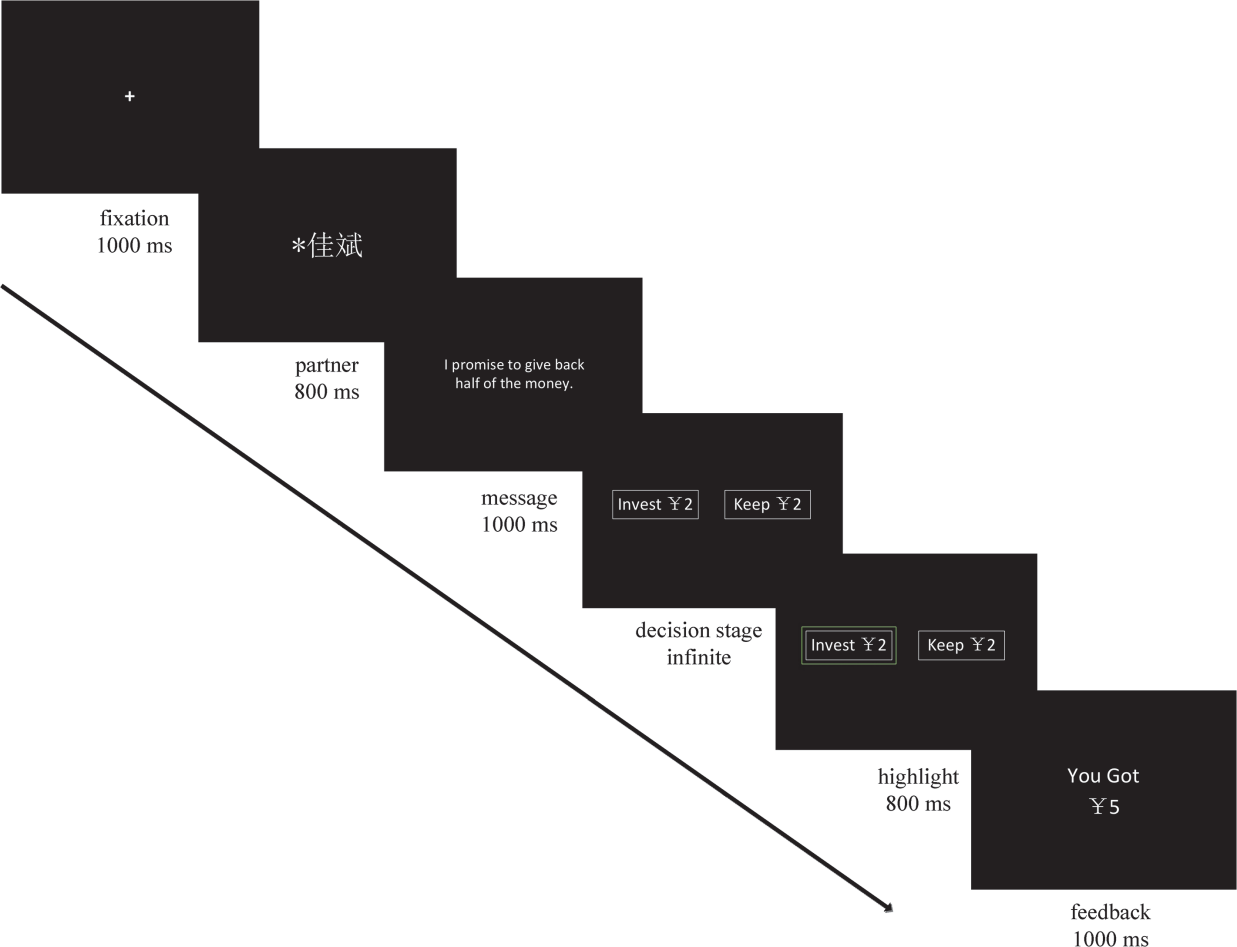
Experimental task. Messages from trustees were shown on the screen. Subjects could either invest to keep the entire tokens endowed on each round. Subject’s earning on that round was displayed if the “invest” option was chosen.

As illustrated in Fig. 1, each trial was initiated by a fixation presented for 1000 ms on the blank screen, which indicated the beginning of each trial. In order to convince the subjects that they were playing with real people instead of computers, the given name of the partner was presented. Then, the partners’ message was presented for 1000 ms. If the partner made a promise, then “I promise to give back half of the money.” would appear on the screen. Otherwise, “The other person did not leave a message.” would be shown. Subsequently, two boxes showing “invest 2” or “keep 2” were presented, and participants were required to choose one of the two boxes by pressing the “1” or “3” key on the keypad. Half of the participants were instructed to press the “1” key if they wanted to invest ¥2, and to press the “3” key if they wanted to keep the money. For the remaining participants, the response pattern was reversed. Once the decision had been made, the chosen box would be highlighted to emphasize the choice. If the participant invested ¥2, after a 800–1000 ms blank screen, a feedback stimulus of either “You got ¥5.” (indicating that the partner returned ¥5 to the participant), or “You got ¥0.” (indicating that the partner kept the entire ¥10) would be presented in the center of the screen for 1000 ms. If the participant did not invest in the partner and kept ¥2, no additional feedback information would be given. Each feedback stimulus was followed by 800 ms of blank screen, and then another trial would start.

To familiarize participants with the task, the experiment started with 4 practice trials. Before the end of the experiment, in order to test whether promise would modulate participants’ expectations toward reciprocity of the partner, they were asked to judge the extent to which they expect their partners to return monetary rewards in both the promise and non-promise conditions using 7-point Likert scale (from 1 = “not likely at all” to 7 = “very likely”). After that, participants were debriefed and paid accordingly.

### EEG Recordings

EEGs were recorded (band-pass 0.05 Hz to 70 Hz, sampling rate 500 Hz) from 64 scalp sites with Neuroscan Synamp2 Amplifier. The left mastoid served as on-line reference. EEGs were off-line re-referenced to the average of the left and the right mastoids. The electrode on the cephalic region was applied as ground. Vertical Electrooculogram (EOG) was recorded supra and infra-orbitally at the left eye, while horizontal EOG was recorded at the left versus right orbital rim. Electrode impedance was maintained below 5 kΩ during the experiment.

### Data Analysis

For the behavioral data, numbers of “invest” and “keep” choices for both promise and non-promise conditions were calculated. Paired t-test was conducted for the comparison of investment rate as well as reciprocity expectation across the two experimental conditions. In addition, two-tailed *Pearson* correlation was carried out between investment rate and reciprocity expectation on the individual level.

For the ERP data, during the offline EEGs analysis, ocular artifacts were removed, which is followed by digital filtering through a zero phase shift (low pass at 30 Hz, 24 dB/octave). Time window of 200 ms before and 800 ms after stimulus presentation was segmented, and the whole epoch was baseline-corrected by the 200 ms interval prior to stimulus onset. Trials containing amplifier clipping, bursts of electromyography activity, or peak-to-peak deflection exceeding ±80 μV were excluded. For each subject, recorded EEGs were separately averaged in each condition over each recording site. Specifically, EEG epochs were separately averaged for outcome (reward/non-reward) × commitment (promise/non-promise) conditions, which resulted in a total of four conditions. Sufficient number of events went into following ERP analysis, with a minimum of 33 valid trials per condition.

Considering that the maximal FRN amplitudes appeared at frontal sites, data from the electrodes F1, Fz, F2, FC1, FCz and FC2 were analyzed. Mean amplitudes in the 260–340 ms time window post-onset of feedback, defined through visual inspection of the d-FRN, went into a 2 (outcome) × 2 (commitment) × 6 (electrode) repeated measures ANOVA. Simple effect analysis was conducted when the interaction effect achieved significance. Greenhouse-Geisser correction was applied in all statistical analyses when necessary.

## Results

### Behavioral Performance

The paired *t*-test showed that participants made more cooperative choices in the promise condition than in the non-promise condition [M_promise_ = 76.21% (SD = 0.11), M_non-promise_ = 61.15% (SD = 0.13), *t* (16) = 3.828, *p* = 0.001]. Furthermore, the behavioral rating for expected reciprocity of partners showed that reward expectation was higher in the promise condition than in the non-promise condition [M_promise_ = 5.24 (SD = 0.90), M_non-promise_ = 3.65 (SD = 1.17), *t* (16) = 4.359, *p* < 0.001]. Correlation analysis was carried out between the two behavioral indicators of investment rate and reciprocity expectation, which were significantly correlated in the promise condition, with a two-tailed *Pearson* correlation level of 0.499 (*p* = 0.041), but not in the non-promise condition (two-tailed *Pearson* correlation = 0.099, *p* = 0.705).

### ERPs

**Fig 2:**
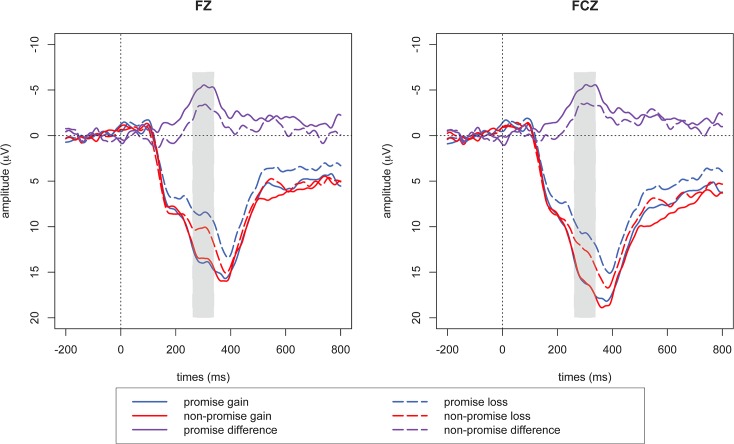
FRN results during outcome evaluation. For illustrative purpose, grand-averaged ERP waveforms of FRN from 2 midline frontal electrodes (Fz, FCz) were shown over outcome (reciprocity vs. non-reciprocity) and commitment (promise vs. non-promise).

Outcome evaluation is mainly reflected in FRN. As presented in Fig. 2, ANOVA analysis for the FRN revealed main effects of outcome (F_1, 16_ = 15.311; *p* = 0.001) and electrode (F_5, 80_ = 15.796; *p* < 0.001), while the main effect of commitment was not significant (F_1, 16_ = 1.044; *p* = 0.322). The interaction effect of outcome and commitment was also significant (F_1, 16_ = 6.805; *p* = 0.019). Data of FCz went into simple effect analysis, because it was reported to show the largest FRN amplitude in most previous literatures [8]. FRN was discrepant between reward and non-reward both in the promise condition (F_1, 16_ = 23.447; *p* < 0.001) and the non-promise condition (F_1, 16_ = 6.042; *p* = 0.026). We further examined the commitment effect in the reward and non-reward conditions respectively. Importantly, this effect was found to be significant in the non-reward condition (F_1, 16_ = 7.873; *p* = 0.013), but not in the reward condition (F_1, 16_ = 0.018; *p* = 0.896).

## Discussion

Long before complex social and legal systems came into existence, promise had become one of the most effective means to establish social contracts in the society, which orally guaranteed the occurrence of certain acts afterwards. Generally speaking, since keeping one’s word is regarded as a potent social norm, promise transfers the information of a person’s trustworthiness. When a promise is given, positive expectation toward that person is generally formed. Thus, when a promise is not fulfilled eventually, it is not only a breach of trust and expectation, but also a violation of one of the most fundamental social norms cherished by the whole society.

In the present study, we adopted the promise-TG to examine the influence of promise on trust and cooperative behavior. During the message-leaving stage, trustees might make non-binding promises of returns contingent on the investment of investors, and investors could decide whether to trust the trustees using promise as a clue. It is worth noticing that promise does not guarantee reciprocation, and the trustee might either honor the promise or take advantage of the device to give a promise first and then break it. This manipulation made it possible for us to examine the influence of promise on investors’ cooperative behaviors as well as their neural responses to the trustees’ promise keeping and promise breaking.

Trust, defined as the willingness to accept vulnerability based on positive expectations about another’s behavior [17], is behaviorally measured as the investment rate in the current experiment. On the aggregate level, investors demonstrated a stronger investment inclination toward trustees who gave promises, which replicated previous findings [1,18]. This indicated that investors expected them to be more likely to return the monetary reward relative to those who did not gave promises. The additional reciprocity expectation rating further supported this interpretation by showing that the level of reward expectation was higher in the promise condition than in the non-promise condition. On the individual level, investors who had higher reciprocity expectation in the promise condition also invested more. Based on these evidences, we can infer that promise is an important tool for trust development in our daily life.

At the neural level, we found a general FRN effect for reciprocity versus non-reciprocity comparison, which is consistent with many previous findings that non-reward outcome elicited a larger deflection of FRN compared with reward outcome [8]. Importantly, promise amplified the size of the FRN effect. Specifically, the modulation effect of promise took place mainly on ERP responses to promise breaking, but not on those to fulfillment of commitment. The augmentation of the FRN effect by promise well extended previous studies, which could be explained by both the reinforcement learning theory and the motivational account of FRN.

In line with the reinforcement learning theory of FRN, in the non-social domain, previous studies have demonstrated that unexpected outcomes would elicit larger FRN effects than expected ones, suggesting that the FRN is sensitive to the prediction error [13,14,19,20]. In the social domain, previous research also showed that violation of the social norm would give rise to larger FRN amplitude [9–11,21]. Although non-binding in nature, compliance with the commitment was generally deemed as a social norm, and failing to do so would be regarded as a severe violation of the norm, which might lead to the commitment effect in the non-reward condition in the current study. To be more specific, promises may change the strength of expectancy toward the positive outcome, which has been verified by the behavioral data. Thus, violation of this stronger expectancy, indicated by non-reward in the promise condition, would give rise to a stronger prediction error, and thus lead to stronger brain responses.

In the present study, promise actually has no predictive value concerning reciprocity of the trustees, since the promise and the non-promise condition have same outcome distributions. Without explicitly varying the objective probability of certain outcomes, the present study suggested that the increased subjective expectancy toward positive outcomes could also influence FRN. In fact, we did find that the enlarged FRN effect following promises was mainly due to more negative-going ERP responses to non-reward, which is consistent with the argument of increased expectancy. Thus, it is reasonable to suggest that expectancy level would modulate individuals’ assessment of the outcome as reflected in the FRN amplitude. Although we did not directly measure investors’ expectations by certain ERP components, but with a behavioral measurement (reciprocation expectation rating) instead, the present study revealed that FRN associated with the feedback outcome might be used as a clue to infer how people would judge other individuals and thus predict others’ behaviors in the social context [22].

The analysis of feedback outcome demonstrated that d-FRN was larger in the promise condition than in the non-promise condition. According to the motivational account of the FRN [14,15,23,24], once the same outcome was attached with enhanced motivational significance, it would lead to stronger FRN responses. In the current study, since investors were more concerned with whether the promise was kept or breached, promise might increase the motivational significance of the outcome, which led to a larger d-FRN in the promise condition.

Previous ERP studies on outcome evaluation have shown that FRN responses to the same feedback could be context-dependent [14,25–27]. For example, in a previous study [14], we asked subjects to perform calculation tasks that required either high effort or low effort, which found that FRN was particularly enhanced when subjects correctly answered the question while did not get a reward in the high effort condition. In the present study, although investors got no reward in both conditions, promise breaking and simply non-reciprocation without giving a promise in advance were differently processed in the brain. Findings in the current research well extended previous studies, indicating that the social context (e.g. social norm) would provide valuable information to outcome evaluation and reward processing.

## Conclusion

Adopting a modified trust game, we investigated the impact of trustees’ promise on investors’ subsequent cooperative behaviors as well as investors’ outcome evaluation resulted from trustees’ actual actions (promise keeping or promise violation). Behaviorally, we found that investors reported a higher degree of return expectation and became more likely to take the investment action when trustees committed to reciprocate. At the neural level, FRN magnitude was prominently enhanced when trustees failed to fulfill the commitment and returned no tokens to the investors, as opposed to the scenario where no promise was given in advance, which might reflect the higher reciprocity expectation induced by the promise. To sum up, the current study complements existing literatures, which proved that cheap talk could boost trust and social cooperation through the augment of investors’ reciprocity expectation, and further modulate the following outcome evaluation in the social context.
